# N-Glycomic Changes in Serum Proteins in Type 2 Diabetes Mellitus Correlate with Complications and with Metabolic Syndrome Parameters

**DOI:** 10.1371/journal.pone.0119983

**Published:** 2015-03-20

**Authors:** Roberto Testa, Valerie Vanhooren, Anna Rita Bonfigli, Massimo Boemi, Fabiola Olivieri, Antonio Ceriello, Stefano Genovese, Liana Spazzafumo, Vincenzo Borelli, Maria Giulia Bacalini, Stefano Salvioli, Paolo Garagnani, Sylviane Dewaele, Claude Libert, Claudio Franceschi

**Affiliations:** 1 Experimental models in Clinical Pathology, Italian National Research Center on Aging (INRCA), Ancona, 60127, Italy; 2 VIB Inflammation Research Center, Technologiepark 927, B-9052, Ghent, Belgium; 3 Department of Molecular Biology, Ghent University, Technologiepark 927, B-9052, Ghent, Belgium; 4 Scientific Direction, Italian National Research Center on Aging (INRCA), Ancona, 60124, Italy; 5 Metabolic Diseases and Diabetology Unit, Italian National Research Center on Aging (INRCA), 60127, Ancona, Italy; 6 Department of Clinical and Molecular Sciences, Università Politecnica delle Marche, Ancona, 60020, Italy; 7 Center of Clinical Pathology and Innovative Therapy, Italian National Research Center on Aging (INRCA), Ancona, 60127, Italy; 8 Institut d'Investigacions Biomèdiques August Pi i Sunyer (IDIBAPS), Barcelona, 08036, Spain; 9 Centro de Investigación Biomédica en Red de Diabetes y Enfermedades Metabólicas Asociadas (CIBERDEM), Barcelona, 08017, Spain; 10 Department of Cardiovascular and Metabolic Diseases, IRCCS Gruppo Multimedica Sesto San Giovanni (MI), 20099, Italy; 11 Center of Biostatistic, Italian National Research Center on Aging (INRCA), Ancona, 60124, Italy; 12 Department of Experimental, Diagnostic and Specialty Medicine Experimental Pathology, University of Bologna, Via S. Giacomo 12, Bologna, 40126, Italy; 13 Center for Applied Biomedical Research, St. Orsola-Malpighi University Hospital, Bologna, 40138, Italy; 14 Interdepartmental Centre "L. Galvani" CIG, University of Bologna, Piazza di Porta S. Donato 1, Bologna, 40126, Italy; 15 IRCCS, Institute of Neurological Sciences of Bologna, Bologna, 40124, Italy; University of Nebraska Medical Center, UNITED STATES

## Abstract

**Background:**

Glycosylation, i.e the enzymatic addition of oligosaccharides (or glycans) to proteins and lipids, known as glycosylation, is one of the most common co-/posttranslational modifications of proteins. Many important biological roles of glycoproteins are modulated by N-linked oligosaccharides. As glucose levels can affect the pathways leading to glycosylation of proteins, we investigated whether metabolic syndrome (MS) and type 2 diabetes mellitus (T2DM), pathological conditions characterized by altered glucose levels, are associated with specific modifications in serum N-glycome.

**Methods:**

We enrolled in the study 562 patients with Type 2 Diabetes Mellitus (T2DM) (mean age 65.6±8.2 years) and 599 healthy control subjects (CTRs) (mean age, 58.5±12.4 years). N-glycome was evaluated in serum glycoproteins.

**Results:**

We found significant changes in N-glycan composition in the sera of T2DM patients. In particular, α(1,6)-linked arm monogalactosylated, core-fucosylated diantennary N-glycans (NG1(6)A2F) were significantly reduced in T2DM compared with CTR subjects. Importantly, they were equally reduced in diabetic patients with and without complications (P<0.001) compared with CTRs. Macro vascular-complications were found to be related with decreased levels of NG1(6)A2F. In addition, NG1(6)A2F and NG1(3)A2F, identifying, respectively, monogalactosylated N-glycans with α(1,6)- and α(1,3)-antennary galactosylation, resulted strongly correlated with most MS parameters. The plasmatic levels of these two glycans were lower in T2DM as compared to healthy controls, and even lower in patients with complications and MS, that is the extreme “unhealthy” phenotype (T2DM+ with MS).

**Conclusions:**

Imbalance of glycosyltransferases, glycosidases and sugar nucleotide donor levels is able to cause the structural changes evidenced by our findings. Serum N-glycan profiles are thus sensitive to the presence of diabetes and MS. Serum N-glycan levels could therefore provide a non-invasive alternative marker for T2DM and MS.

## Introduction

Type 2 diabetes mellitus (T2DM) is a complex and heterogeneous disease with a strong genetic propensity when linked to a typical Western lifestyle, however, apart from this fact, its etiology is still poorly understood. It is characterized by a chronic hyperglycemia, insulin resistance, and a relative insulin secretion defect. Obesity and sedentary lifestyles correlate with T2DM and its diffusion, however many aspect of involved biochemical pathways are still poorly known [1]. Indeed there are many biochemical alterations other than hyperglycemia characterizing T2DM that lack a physio-pathological mechanism. It is now clear that T2DM and other condition with minor degrees of glucose intolerance commonly occur together with a collection of clinical and biochemical features, that have been called metabolic syndrome [2–4].

The term “metabolic syndrome” (MS) defines a cluster of components that reflect over nutrition, sedentary lifestyles and resultant excess adiposity. It melts together a cluster of cardiovascular risk factors whose core components are: impaired glucose metabolism, obesity, dyslipidemia, and hypertension. MS is also associated with other co-morbidities, such as prothrombotic state, proinflammatory state, nonalcoholic fatty liver disease and reproductive disorders. The prevalence of the MS is growing to epidemic proportions all over the world [5], both in the urbanized world and in developing nations. Although there are divergent criteria for the identification of the MS [6,7] there is current agreement that obesity [waist circumference (WC)], insulin resistance, dyslipidemia and hypertension [8] are MS core components. Moreover, MS is strictly related to T2DM with concomitant cardiovascular diseases (CVD) [9].

N-linked oligosaccharides of glycoproteins (N-glycans) are emerging as powerful and reliable biomarkers of several diseases [10]. N-glycans play important biological roles by influencing the functions of glycoprotein [11] involved in various cellular recognition signals. N-glycans are also involved in pathological situations such as cancer and inflammation [12–15]. Usage of N-glycans as biomarkers in clinical practice is facilitated by the existence of methodology such as high-throughput technology platform designed to profile N-glycans on proteins (DSA-FACE) [16].

It is known that an aberrant O-GlcNAc modification of proteins is involved in T2DM, as well as in cardiovascular diseases and insulin resistance [17–20]. It is hypothesized that in these pathological conditions hyperglycemia causes an increase in the levels of UDP-*N-*acetylglucosamine (UDP-*N*-GlcNAc) through the hexosamine biosynthetic pathway (HBP). In such a pathway, UDP-GlcNAc is the main sugar donor substrate for O-GlcNAc transferase (OGT), an enzyme that catalyzes a reversible form of post-translational protein O-glycosylation [21, 22]. At variance, little is known regarding the changes in N-glycans during MS and T2DM. In particular, a study conducted on mice with T2DM demonstrated an increase of core-fucosylated serum N-glycans and, concomitantly, increased mRNA levels of α-1,6-fucosyltransferase in the liver [23]. It has been demonstrated that modifications of fucose content in serum glycoproteins occur also in T2DM patients [24–25] together with a deficit on GnT-4a glycosyltransferase activity in their pancreatic beta cells [26], i.e the enzyme that generates the core β1–4 GlcNAc linkage among the N-glycan structures.

The biosynthesis of glycans depends on the complicated concerted action of glycosyltransferases, therefore the structures of glycans are much more variable than those of proteins and nucleic acids. N-glycan synthesis can be easily altered by pathophysiological conditions [10] such as inflammatory and autoimmune diseases and in the pathophysiological process of aging. Accordingly, glucose-related alterations of the glycans could be relevant to understand the complex physiological changes in metabolic syndrome and diabetes mellitus. Therefore we determined the changes in N-glycome on serum glycoproteins in a large cohort of healthy subjects and Type 2 diabetic subjects with or without metabolic syndrome.

## Materials and Methods

### Patients

Five hundred and sixty two T2DM patients (mean age (SD), 65.6 (8.2) years) and 599 healthy control subjects (CTRs) (mean age (SD), 58.5 (12.4) years) have been enrolled from central part of Italy after informed consent was obtained from each subject. The study protocol was approved by the Ethics Committee of INRCA. All subjects gave written informed consent. T2DM was diagnosed according to the American Diabetes Association Criteria [27]. Inclusion criteria were: BMI <40 kg/m2, age 35 to 85 years, ability and willing to give written informed consent and to comply with the requirements of the study. Information collected included data on vital signs, anthropometric factors, medical history and behaviors, as well as physical activity. The presence/absence of diabetic complications was evidenced as follows: diabetic retinopathy by fundoscopy through dilated pupils and/or fluorescence angiography; incipient nephropathy, defined as an urinary albumin excretion rate >30 mg/24h and a normal creatinine clearance; renal failure, defined as an estimated glomerular filtration rate >60 mL/min per 1.73 m^2^; neuropathy established by electromyography; ischemic heart disease defined by clinical history, and/or ischemic electrocardiographic alterations; peripheral vascular disease including atherosclerosis obliterans and cerebrovascular disease on the basis of history, physical examinations and Doppler velocimetry. Among the 309 T2DM patients with at least one complication, 102 were affected by neuropathy, 35 by lower limb arteriopathy obliterans, 25 by arteriopathy of the super-aortic trunks, 96 by cardiovascular ischemia, 71 by nephropathy, 150 by retinopathy and 21 by renal failure. Hypertension was defined as a systolic blood pressure >140 mmHg and/or a diastolic blood pressure >90 mmHg, measured while the subjects were sitting, which was confirmed in at least three different occasions. All the selected subjects consumed a Mediterranean diet. The diagnosis of metabolic syndrome was made depending on the presence of at least 3 of the following parameters, according to Adult Treatment Panel III-2001 (ATP III) criteria: abdominal obesity (WC > 102 cm for males and > 88 cm for females), hypertension (systolic blood pressure > 130 mm Hg and/or diastolic blood pressure > 85 mm Hg) or history of antihypertensive usage, hypertriglyceridemia (≥ 150 mg/dl) or presence of treatment for this disorder, low HDL-C (< 40 mg/dl in males and < 50 mg/ dl in females), and high fasting plasma glucose (≥ 110 mg/dl) or presence of diagnosis of T2DM [6,28]. Overnight fasting venous blood samples of all subjects were collected from 8:00 to 9:00 a.m. The samples were either analyzed immediately or stored at -80°C for no more than 10 days.

### Laboratory assays

Blood concentrations of total and HDL cholesterol, triglycerides, fasting glucose, HbA1c, fasting insulin, and WBC were measured by standard procedures.

### N-glycan analysis using the ABI 3130 sequencer

The N-linked glycans present on the serum proteins were analyzed using DSA-FACE technology (as described in [16, 29]. Briefly, the glycoproteins were denatured by adding 2 μL of denaturing buffer (10 mM NH_4_HCO_3_, pH 8.3, 5% SDS) to 2 μL serum in a PCR 96-well plate. The plate was heated at 95°C for 5 min and cooled for 15 min in a PCR thermocycler, then combined with 3 μL of peptide-Nglycosidase F (PNGase F; 2.2U/uL and 3.33% NP40 in denaturing buffer, New England Biolabs). The plate was incubated at 37°C for 3 h. Subsequently, 100 uL of water was added, and 6 μL of the resulting solution was transferred to a new PCR plate and evaporated to dryness at 60°C in the thermocycler. N-Glycans were derivatized by adding 2 μL of a labeling solution (1:1 mixture of 20 mM 8-Amino-1,3,6-PyreneTriSulfonic acid (APTS, Molecular Probes) in 1.2 M citric acid and 1 M NaCNBH_3_ in dimethyl sulfoxide). The tightly closed plate was then heated at 37°C for 16 h. Water (200 μL) was added to stop the reaction and to dilute the label to approximately 100 pmol/μL. In order to separate the N-glycans according to size and not to charge, the glycan sialic acids groups, containing negative charges, are removed by a sialidase-digestion. Two μL of the solution was transferred to a new 96 well plate. Digestion with 0.2 μL of *Arthrobacter ureafaciens* sialidase (Roche Diagnostics) was done in 3 μL of 20 mM NH_4_Ac, pH 5, and the plate was incubated 16 h at 37°C. Lastly, was added 160 μL of water. 10 ul of these labeled N-glycans were analyzed by DSA-FACE technology, using an ABI 3130 sequencer (Applied Biosystems). Data analysis was performed using the Genescan 3.1 software (Applied Biosystems).

### Serum Protein N-Glycan Profiling

At last 10 peaks were detected in all the samples, with each peak representing a different N-glycan structure (S1 Fig.).

In particular: peak 1 is an agalacto, core-α-1,6-fucosylated diantennary glycan (NGA2F); peak 2 is an agalacto core-α-1,6-fucosylated bisecting diantennary glycan (NGA2FB); peak 3 and peak 4 assess the isomers arising from upper α(1,6)- *vs* lower (a1,3)-arm galactosylation of the core-α-1,6-fucosylated diantennary glycan NG1A2F, *i*.*e*, respectively, the isomer α(1,6)-arm monogalactosylated, NG1(6)A2F, and the isomer α(1,3)-arm monogalactosylated, NG1(3)A2F; peak 5 is a digalacto diantennary glycan (NA2); peak 6 is a digalacto core-α-1,6-fucosylated diantennary glycan (NA2F); peak 7 is a digalacto core α-1,6-fucosylated bisecting diantennary glycan (NA2FB), peak 8 is a triantennary glycan (NA3), peak 9 is a branching α-1,3-fucosylated triantennary glycan (NA3F), peak 10 (P10) is a tetra-galactosylated core-α-1,6-fucosylated tetrantennary glycan (NA4). The structures of peaks as well as the isomers distribution of NG1A2F have been assigned as previously indicated by Liu *et al*. [30] and Bunz *et al*. [31].

We quantified the heights of the ten peaks that were detected in all the samples to obtain a numerical description of the profiles, and analyzed these data with SPSS 17.0.

### Statistical analysis

Data were analyzed with R. Robust linear regression was performed using the package *Robustbase* (CIT). The package *multtest* was used to perform the False Discovery Rate (FDR) correction for multiple comparisons using the Benjamini–Hochberg procedure. Pearson partial correlation coefficients were calculated to analyze the association between peaks values and the other independent variables.

## Results

### Comparison of serum N-glycans profiles in T2DM, T2DM without complications (T2DM-), and T2DM with complication (T2DM+)

We examined the N-glycome profile of desialylated serum from a cohort of 1162 samples, characterized for the presence of diabetes, diabetic complications and metabolic syndrome (Table 1).

**Table 1:** Characteristics of the cohort under analysis.

Presence of diabetes	Gender	Presence/Absence of complications	Presence/Absence of MS
	Mean age ± SD	Mean age ± SD	Mean age ± SD
**Controls**	Males N = **231**	-	With MS: N = **21**
			(61.8±12.7)
	(58.8±11.7)		Without MS N = **210**
			(58.5±11.6)
	Females N = **368**	-	With MS N = **49**
			(65.3±10.4)
	(58.2±12.7)		Without MS N = **319**
			(57.1±12.7)
**Diabetic patients**	Males N = **302**	With complications N = **189**	With MS N = **92**
			(66.2±7.2)
		(65.6±8.1)	Without MS N = **97**
			(65.0±8.8)
	(65.0±8.3)	Without complications N = **113**	With MS N = **51**
			(63.3±7.8)
		(64.1±8.5)	Without MS N = **62**
			(64.7±9.0)
	Females N = **260**	With complications N = **120**	With MS N = **91**
			(68.8±6.5)
		(68.9±6.6)	Without MS N = **29**
			(69.2±7.1)
	(66.6±7.8)	Without complications N = **140**	With MS N = **100**
			(64.7±8.1)
		(64.7±8.1)	Without MS N = **40**
			(64.7±8.4)

Subjects were classified on the basis of the presence of diabetes, diabetic complications and metabolic syndrome (MS). For each class, the number(N) of males and females is indicated, while the mean age ± it's standard deviation(SD) are reported between round brackets.

As it is well established that sex influences the serum N-glycosylation pattern (see in S1 Table), males and females were analyzed separately.

A robust linear model was built for each peak and age was used as covariate in order to correct for age-dependent variations in N-glycan peaks. We first focused on N-glycans features whose abundance was significantly different between CTR and T2DM, independently from the presence of complications (Table 2).

**Table 2:** Multiple comparison of serum N-glycans changes between diabetic patients; diabetic patients with/without complications and controls.

	CTR	T2DM	T2DM-	T2DM+	T2DM+ vs T2DM-
	Mean (SD)	Mean (SD)	Mean (SD)	Mean (SD)	
		q-value	q-value	q-value	q-value
Males	N = 231	N = 302	N = 113	N = 189	
Peaks (structure)					
**P1** (NGA2F)	9.36 (2.59)	9.69 (3.23)	9.63 (3.39)	9.73 (3.14)	
					*ns*
**P2** (NGA2FB)	1.59 (0.49)	1.84 (0.69)	1.78 (0.58)	1.87 (0.74)	
					*ns*
**P3** (NG1(6)A2F)	6.16 (1.29)	5.18 (1.36)	5.34 (1.39)	5.09 (1.34)	
		***<0*.*001***	***<0*.*001***	***<0*.*001***	*ns*
**P4** (NG1(3)A2F)	5.08 (0.82)	4.81 (1.01)	4.88 (0.91)	4.78 (1.07)	
		***<0*.*01***		***<0*.*01***	*ns*
**P5** (NA2)	43 (3.99)	44.25 (4.85)	43.97 (4.97)	44.42 (4.79)	
		***<0*.*01***		***<0*.*01***	*ns*
**P6** (NA2F)	18.03 (2.49)	17.45 (2.52)	17.49 (2.41)	17.43 (2.59)	
					*ns*
**P7** (NA2FB)	5.89 (1.29)	6.19 (2)	6.03 (1.65)	6.28 (2.18)	
					*ns*
**P8** (NA3)	6.64 (1.82)	6.39 (1.74)	6.68 (1.74)	6.21 (1.72)	
					*ns*
**P9** (NA3F)	2.81 (1.24)	2.86 (1.05)	2.83 (1.02)	2.88 (1.06)	
					*ns*
**P10** (NA4)	1.44 (0.49)	1.34 (0.43)	1.37 (0.45)	1.31 (0.41)	
					*ns*

Females	N = 368	N = 260	N = 140	N = 120	
Peaks (structure)					
**P1** (NGA2F)	9.12 (3.17)	9.91 (3.04)	9.77 (3.18)	10.07 (2.87)	
					*ns*
**P2** (NGA2FB)	1.64 (0.65)	1.93 (0.67)	1.88 (0.71)	2 (0.63)	
					*ns*
**P3** (NG1(6)A2F)	5.82 (1.33)	5.01 (1.33)	5.09 (1.39)	4.91 (1.25)	
		***<0*.*001***	***<0*.*001***	***<0*.*001***	*ns*
**P4** (NG1(3)A2F)	4.9 (0.91)	4.54 (0.74)	4.58 (0.75)	4.48 (0.73)	
		***<0*.*01***			*ns*
**P5** (NA2)	43.44 (4.34)	44.01 (4.37)	43.93 (4.85)	44.1 (3.74)	
					*ns*
**P6** (NA2F)	17.6 (2.99)	16.74 (2.4)	16.88 (2.54)	16.58 (2.22)	
					*ns*
**P7** (NA2FB)	6.07 (1.43)	6.16 (1.52)	6.13 (1.54)	6.2 (1.51)	
					*ns*
**P8** (NA3)	7.59 (1.81)	7.8 (1.87)	7.91 (1.95)	7.66 (1.78)	
					*ns*
**P9** (NA3F)	2.14 (1)	2.23 (1.09)	2.18 (1.09)	2.29 (1.09)	
					*ns*
**P10** (NA4)	1.67 (0.5)	1.69 (0.47)	1.66 (0.46)	1.72 (0.48)	
					*ns*

For each peak the mean and the SD are indicated. The reference group in each comparison is CTR. For each comparison, the FDR-corrected p-value (q-value) is reported if <0.05 Structure abbreviations: N-glycans (N) have a common pentasaccharide core denoted as A0 and consists of two N-acetylglucosamines (GlcNAc) and three mannose residues; F α-(1–6) linked core fucose; Ax: number of antennary GlcNAc attached to the trimannosyl core; B: bisecting GlcNAc; Gx: number of α(1–4) linked galactose (G); G1(3) and G1(6) indicates that the galactose is either on the α(1–3) or α(1–6) antenna.

Both in males and in females, the α(1,6)-arm monogalactosylated, NG1(6)A2F, as well as the α(1,3)-arm monogalactosylated, NG1(3)A2F, core-α-1,6-fucosylated diantennary glycans (respectively identified by peak 3 and peak 4), resulted significantly lower in T2DM respect to CTR (NG1(6)A2F, *P* <0.001; NG1(3)A2F, *P* <0.01). In addition, digalactosylated diantennary glycans NA2, (or peak 5) were significantly higher in T2DM respect to CTR, but only in male patients (*P* <0.01).

Then, we compared T2DM- and T2D2M+ with CTR. We found that both in males and in females NG1(6)A2F glycans were significantly lower in T2DM- (NG1(6)A2F, *P*<0.001) and in T2DM+ (*P*<0.001) respect to CTR. Notably, the decrease in NG1(6)A2F levels was more evident in T2DM+, while T2DM- showed intermediate values between CTR and T2DM+. In males, but not in females, NG1(3)A2F and NA2 N-glycans resulted significantly different between T2DM+ and CTR, although also in this case the levels of the two peaks in T2DM- was halfway. A similar trend was observed for NG1(3)A2F and NA2 in females, although it did not reach statistical significance. No significant differences were found when we compared T2DM- with T2DM+.

### Association of serum N-glycans with diabetic complications

We then evaluated the association between serum N-glycomic peaks values and diabetic complications. To this aim, we grouped diabetic patients with complications (i.e. T2DM+) in 2 new groups: i) T2DM+*, including diabetic patients with micro-complications (i.e patients affected by neuropathy or/and nephropathy, or/and renal failure, or/and retinopathy), and ii) T2DM+**, including diabetic patients with macro-complications (i.e. patient with at least one of these complications: lower limb arteriopathy obliterans, arteriopathy of the super aortic trunks, cardiovascular diseases). We compared the levels of each peak between T2DM-, T2DM+* and T2DM+**, subdividing the cohort on the basis of the sex of the subjects and using age as covariate (Table 3).

**Table 3:** Multiple comparisons between diabetic patients without complications and diabetic patients with micro and macro-complications.

	T2DM- without complication	T2DM+* with micro complications	T2DM+** with macro complications	T2DM+* vs T2DM+**
	Mean(SD)	Mean(SD)	Mean(SD)	
	*P value*	*P value*	*P value*	*P value*
Males	N = 113	N = 101	N = 88	
**Peaks (structure)**				
**P1** (NGA2F)	9.63 (3.39)	9.89 (3.38)	9.54 (2.84)	
				*ns*
**P2** (NGA2FB)	1.78 (0.58)	1.84 (0.88)	1.9 (0.55)	
				*ns*
**P3** (NG1(6)A2F)	5.34 (1.39)	5.28 (1.37)	4.86 (1.29)	
			***P = 0*.*01***	*ns*
**P4** (NG1(3)A2F)	4.88 (0.91)	4.87 (1.2)	4.67 (0.9)	
				*ns*
**P5** (NA2)	43.97 (4.97)	44.28 (5.02)	44.58 (4.53)	
				*ns*
**P6** (NA2F)	17.49 (2.41)	17.38 (2.83)	17.47 (2.3)	
				*ns*
**P7** (NA2FB)	6.03 (1.65)	5.97 (1.8)	6.65 (2.5)	
				*ns*
**P8** (NA3)	6.68 (1.74)	6.29 (1.69)	6.13 (1.77)	
				*ns*
**P9** (NA3F)	2.83 (1.02)	2.85 (1.14)	2.91 (0.97)	
				*ns*
**P10** (NA4)	1.37 (0.45)	1.33 (0.4)	1.29 (0.43)	
				*ns*

**Females**	N = **140**	N = **71**	N = **49**	
**Peaks (structure)**				
**P1** (NGA2F)	9.77 (3.18)	9.95 (2.77)	10.24 (3.02)	
				*ns*
**P2** (NGA2FB)	1.88 (0.71)	1.99 (0.6)	2 (0.67)	
				*ns*
**P3** (NG1(6)A2F)	5.09 (1.39)	5.04 (1.18)	4.73 (1.33)	
			***P = 0*.*05***	*ns*
**P4** (NG1(3)A2F)	4.58 (0.75)	4.51 (0.65)	4.44 (0.84)	
			***P = 0*.*02***	*ns*
**P5** (NA2)	43.93 (4.85)	43.8 (3.42)	44.53 (4.17)	
				*ns*
**P6** (NA2F)	16.88 (2.54)	16.99 (2.14)	15.98 (2.22)	
				***P = 0*.*01***
**P7** (NA2FB)	6.13 (1.54)	6.42 (1.62)	5.87 (1.28)	
				*ns*
**P8** (NA3)	7.91 (1.95)	7.39 (1.78)	8.05 (1.72)	
				***P<0*.*01***
**P9** (NA3F)	2.18 (1.09)	2.22 (1.07)	2.38 (1.13)	
				*ns*
**P10** (NA4)	1.66 (0.46)	1.68 (0.45)	1.77 (0.51)	
				*ns*

For each peak the mean and the SD are indicated. The reference group in each comparison is T2DM-. For each comparison the nominal p-value is reported if < = 0.05.

Although after FDR correction no comparison reached the statistical significance, NG1(6)A2F levels differed between T2DM+** and T2DM- (*P* = 0.01 in males and *P* = 0.05 in females), but not between T2DM+* and T2DM-. In females, but not in males, minor differences were observed also for NG1(3)A2F (*P* = 0.02) between T2DM+** and T2DM- and for the digalacto core-fucosylated diantennary glycans (NA2F) and the triantennary glycans (NA3) between T2DM+** and T2DM+* (*P* = 0.01 and *P*<0.01 respectively).

### Association of serum N-glycans with metabolic syndrome

As reported in Table 1, metabolic syndrome affects not only a large fraction of diabetic patients from our cohort, but also a number of non-diabetic controls. Based on these considerations, we compared the N-glycomic profiles of healthy controls (not affected by diabetes or MS) with those of T2DM patients with or without complications and with or without MS (Table 4).

**Table 4:** Serum N-glycans differences between controls and diabetic patients with and without MS.

	CTR without MS	CTR with MS	T2DM- without MS	T2DM- with MS	T2DM+ without MS	T2DM+ with MS
	Mean(SD)	Mean(SD)	Mean(SD)	Mean(SD)	Mean(SD)	Mean(SD)
	*P value*	*P value*	*P value*	*P value*	*P value*	*P value*
Males	N = 210	N = 21	N = 113	N = 62	N = 97	N = 92
Peaks (structure)						
**P1** (NGA2F)	9.28 (2.58)	10.21 (2.61)	9.29 (2.66)	10.04 (4.09)	9.82 (3.16)	9.63 (3.13)
**P2** (NGA2FB)	1.59 (0.5)	1.6 (0.38)	1.72 (0.54)	1.85 (0.63)	1.88 (0.81)	1.86 (0.68)
**P3** (NG1(6)A2F)	6.2 (1.32)	5.7 (0.88)	5.38 (1.35)	5.3 (1.45)	5.3 (1.28)	4.87 (1.38)
			***P<0*.*01***	***P<0*.*001***	***P<0*.*001***	***P<0*.*001***
**P4** (NG1(3)A2F)	5.09 (0.83)	4.9 (0.8)	5.03 (1)	4.68 (0.75)	4.88 (0.8)	4.67 (1.3)
				***P<0*.*05***		***P<0*.*001***
**P5** (NA2)	42.97 (4.06)	43.31 (3.23)	44.15 (4.51)	43.75 (5.51)	44.09 (4.69)	44.77 (4.89)
						***P<0*.*01***
**P6** (NA2F)	18.11 (2.48)	17.26 (2.52)	17.78 (2.21)	17.14 (2.62)	17.62 (2.53)	17.22 (2.65)
**P7** (NA2FB)	5.92 (1.32)	5.6 (0.93)	6.04 (1.41)	6.02 (1.91)	6.35 (1.76)	6.22 (2.55)
**P8** (NA3)	6.57 (1.79)	7.29 (1.99)	6.52 (1.66)	6.88 (1.82)	6.03 (1.73)	6.41 (1.7)
**P9** (NA3F)	2.84 (1.24)	2.52 (1.26)	2.74 (1.02)	2.94 (1.02)	2.74 (1.09)	3.02 (1.02)
**P10** (NA4)	1.42 (0.49)	1.62 (0.52)	1.35 (0.43)	1.41 (0.48)	1.3 (0.43)	1.33 (0.39)

Females	N = 319	N = 49	N = 40	N = 100	N = 29	N = 91
Peaks (structure)						
**P1** (NGA2F)	8.9 (3)	10.61 (3.8)	9.4 (3.4)	9.91 (3.1)	9.69 (2.46)	10.19 (2.99)
**P2** (NGA2FB)	1.58 (0.64)	2 (0.58)	1.68 (0.62)	1.96 (0.72)	1.93 (0.64)	2.02 (0.63)
**P3** (NG1(6)A2F)	5.92 (1.32)	5.21 (1.3)	5.43 (1.67)	4.95 (1.25)	4.88 (1.44)	4.92 (1.19)
		***P = 0*.*05***		***P<0*.*001***	***P<0*.*01***	***P<0*.*001***
**P4** (NG1(3)A2F)	4.95 (0.91)	4.61 (0.86)	4.71 (0.86)	4.53 (0.71)	4.57 (0.73)	4.45 (0.73)
				***P<0*.*01***		***P<0*.*01***
**P5** (NA2)	43.33 (4.24)	44.13 (4.9)	43.45 (5.62)	44.12 (4.52)	45.11 (3.51)	43.78 (3.77)
**P6** (NA2F)	17.93 (2.96)	15.47 (2.22)	17.48 (3.18)	16.64 (2.21)	16.39 (1.55)	16.64 (2.4)
		***P<0*.*01***				
**P7** (NA2FB)	6.14 (1.4)	5.6 (1.52)	6.28 (1.54)	6.07 (1.55)	6.02 (1.52)	6.25 (1.51)
		***P = 0*.*01***				
**P8** (NA3)	7.49 (1.71)	8.22 (2.31)	7.78 (1.59)	7.96 (2.08)	7.19 (1.97)	7.81 (1.7)
**P9** (NA3F)	2.12 (0.97)	2.26 (1.17)	2.18 (0.93)	2.18 (1.16)	2.65 (0.93)	2.17 (1.12)
**P10** (NA4)	1.64 (0.45)	1.88 (0.71)	1.62 (0.37)	1.68 (0.49)	1.55 (0.48)	1.77 (0.47)

For each peak the mean and the SD are indicated. The reference group in each comparison is CTR without MS. For each comparison, the FDR-corrected p-value (q-value) is reported if <0.05.

**Fig 1:**
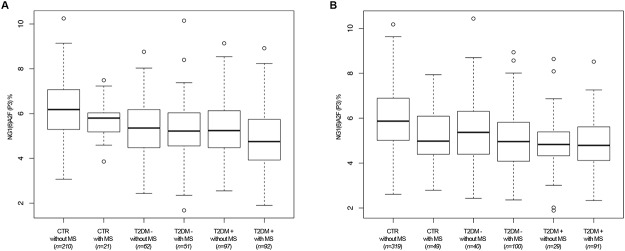
1A. The abundance of α(1,6)-arm monogalactosylated, core-α-1,6-fucosylated diantennary glycan NG1(6)A2F, assessed by peak 3 (P3) levels, in CTR and T2DM patients with and without MS. The boxplots represent a comparison of peak 3 levels in males in six classes of subjects: CTR without MS, CTR with MS, T2DM- without MS, T2DM- with MS, T2DM+ without MS, T2DM+ with MS. **1B.** The abundance of α(1,6)-arm monogalactosylated, core-α-1,6-fucosylated diantennary glycan NG1(6)A2F, assessed by peak 3 (P3) levels, in CTR and T2DM patients with and without MS. The boxplots represent a comparison of peak 3 levels in females in six classes of subjects: CTR without MS, CTR with MS, T2DM- without MS, T2DM- with MS, T2DM+ without MS, T2DM+ with MS.

Interestingly, in males NG1(6)A2F followed a decreasing trend from the extreme “healthy” phenotype (CTR without MS) to the extreme “unhealthy” phenotype (T2DM+ with MS) (Fig. 1).

This trend was present, although in a less regular fashion, also in females. In both males and females, NG1(3)A2F resulted significanly lower in T2DM respect to CTR only when the patients were affected also by metabolic syndrome, suggesting a synergistic effect of the two pathological conditions in altering the peak levels. Finally, in females digalacto core-α-1,6-fucosylated diantennary glycans (NA2F) and digalacto core α-1,6-fucosylated bisecting diantennary glycans (NA2FB)) were specifically altered in CTR affected by MS (Table 4).

### Correlation of N-glycans profiling with metabolic syndrome parameters

Finally, we evaluated the correlation of metabolic syndrome parameters with the glycosylation features. Person correlation was calculated using the entire cohort, subdivided only on the basis of the sex of the subjects (533 males and 628 females; Table 5).

**Table 5:** Pearson correlation of serum N-glycans peaks with metabolic syndrome parameters.

	BMI	Waist.H/P	Glic	HbA1c	NEU ABS	HDL	Trigl	Insulin	HOMA
Males									
**Peaks (structure)**									
**P1** (NGA2F)	0.04	0	0.02	0.01	0.04	-0.01	0	-0.01	0.04
**P2** (NGA2FB)	0.09	0.11	**0.16**	0.15	0.03	-0.06	0.04	-0.04	0.04
		<0.05	**<0.01**	<0.01					
**P3** (NG1(6)A2F)	-0.08	**-0.24**	**-0.32**	**-0.33**	**-0.23**	0.06	**-0.14**	-0.01	**-0.19**
		**<0.001**	**<0.001**	**<0.001**	**<0.001**		**<0.01**		**<0.001**
**P4** (NG1(3)A2F)	-0.06	**-0.18**	**-0.16**	**-0.15**	**-0.15**	0.09	-0.11	-0.08	-0.11
		**<0.001**	**<0.01**	**<0.01**	**<0.01**		<0.05		<0.05
**P5** (NA2)	0.02	0.1	0.13	**0.17**	0.13	-0.02	-0.01	-0.01	0.07
			<0.05	**<0.01**	<0.05				
**P6** (NA2F)	-0.02	-0.11	-0.08	-0.08	**-0.2**	0.03	-0.01	0.05	-0.03
		<0.05			**<0.001**				
**P7** (NA2FB)	-0.05	0.06	0.06	0.02	-0.06	0.02	0.04	0.05	0.09
**P8** (NA3)	0.04	0.04	0	-0.03	0.06	-0.07	**0.16**	0	-0.05
							**<0.01**		
**P9** (NA3F)	-0.01	0.07	-0.03	-0.01	**0.19**	0.01	-0.02	-0.01	-0.02
					**<0.001**				
**P10** (NA4)	-0.02	-0.02	-0.05	-0.08	0	-0.05	0.04	-0.07	-0.08

**Females**									
**Peaks (structure)**									
**P1** (NGA2F)	**0.16**	0.22	0.12	0.11	-0.01	-0.05	0.08	0.1	0.13
	**<0.001**		<0.01	<0.01				<0.05	<0.01
**P2** (NGA2FB)	0.18	0.29	0.23	0.21	0.05	-0.09	**0.16**	0.14	**0.19**
						<0.05	**<0.001**	<0.001	**<0.001**
**P3** (NG1(6)A2F)	**-0.17**	**-0.21**	**-0.32**	**-0.35**	**-0.22**	0.11	**-0.24**	-0.09	**-0.25**
	**<0.001**	**<0.001**	**<0.001**	**<0.001**	**<0.001**	<0.05	**<0.001**	<0.05	**<0.001**
**P4** (NG1(3)A2F)	-0.1	**-0.21**	**-0.25**	**-0.24**	**-0.19**	**0.2**	**-0.21**	**-0.17**	-0.14
	<0.05	**<0.001**	**<0.001**	**<0.001**	**<0.001**	**<0.001**	**<0.001**	**<0.001**	<0.001
**P5** (NA2)	0.01	0.06	0.07	0.09	0.11	-0.03	0.04	0	0.02
				<0.05	<0.01				
**P6** (NA2F)	**-0.19**	**-0.33**	-0.13	**-0.17**	-0.11	0.1	**-0.16**	-0.1	-0.07
	**<0.001**	**<0.001**	<0.01	**<0.001**	<0.01	<0.5	**<0.001**	<0.05	
**P7** (NA2FB)	-0.12	-0.08	0.02	-0.02	-0.05	0.07	-0.05	-0.13	-0.06
	<0.01							<0.01	
**P8** (NA3)	0.1	0.12	0.09	0.14	0.11	**-0.15**	**0.26**	0.12	0.08
	<0.05	<0.05	<0.05	<0.001	<0.05	**<0.001**	**<0.001**	<0.01	
**P9** (NA3F)	0.1	0.12	0	0	0.09	-0.02	-0.04	0.09	-0.01
	<0.05	<0.01			<0.05			<0.05	
**P10** (NA4)	0.07	0.06	0.05	0.1	0.11	-0.13	**0.18**	0.08	0.02
				<0.05	<0.01	<0.01	**<0.001**		

Significant results are highlighted in bold considering a correlation coefficient cut off value of 0.15 and a P = <0.05.

Interestingly, considering an effect size >0.15 and a *P*<0.05, we found that both in males and in females NG1(6)A2F and NG1(3)A2F levels were strongly negatively correlated with most of metabolic syndrome parameters, including waist/hip ratio, triglycerides, glycemia, glycated haemoglobin and the absolute number of neutrophils. In addition, NG1(6)A2F levels were negatively correlated with HOMA. In females, a negative correlation was found between NG1(6)A2F and BMI, between NG1(3)A2F and insulin levels and between NG1(6)A2F and NG1(3)A2F and triglyceride levels. Consistently, NG1(3)A2F was positively correlated with HDL levels. Although NG1(6)A2F and NG1(3)A2F were the main N-glycans that emerged from this analysis, other peaks, including P2 (agalacto core-α-1,6-fucosylated bisecting diantennary glycans (NGA2FB), digalacto core-α-1,6-fucosylated diantennary glycans (NA2F), tri-(NA3) and tetragalactosylated (NA4) glycans resulted significantly associated with one or more metabolic syndrome parameters.

## Discussion

Glycosylation is the enzymatic addition of oligosaccharides (also known as glycans) to proteins and lipids, and it is one of the most common co-/posttranslational modifications of proteins. Most of the human secreted and membrane-bound proteins are glycosylated, suggesting a determinant role of carbohydrates in protein function [32]. Moreover altered glycosylation characterized by different number (macroheterogeneity) and nature of glycans (microheterogeneity) [25] is present in many pathophysiological conditions such as cancer, inflammation, autoimmune and aging [11, 14]. Thus, it is a dynamic equilibrium. Within an individual, the glycan signature is highly reproducible [33], however, during aging or pregnancy or when a disease occurs the glycan pattern can change dramatically [34, 35].

An N-glycan (N-linked oligosaccharide) is a sugar chain covalently linked to an asparagine residue of a polypeptide chain. This link occurs usually through a N-acetylglucosamine (GlcNAc) residue that is bound to a consensus peptide sequence: Asn-XSer. Furthermore, a GlcNAc sugar residue can be attached in O-linkage (O-GlcNAc) to specific Ser/Thr residues of proteins. This relatively recently identified form of nucleocytoplasmic O-glycosylation is mediated by O-GlcNAc transferase (OGT) and O-GlcNAcase (OGA) [36]. Recent studies demonstrated that high levels of circulating glucose, diabetes and diabetic complications are linked with this type of post-translational modification [37–39].

We have used DSA-FACE based N-glycan analysis system to quantify and profile N-glycosilaton of human serum proteins. This high-throughput technology is robust, reproducible, sensitive, and, importantly, quantitative [40]. We found that that α(1,6)-arm monogalactosylated core-α-1,6-fucosylated diantennary glycans, NG1(6)A2F, (discriminated by peak 3 were clearly altered in patient with type 2 diabetes. In particular NG1(6)A2F levels resulted significantly lower in all (with and without complication) T2DM respect to CTR (NG1(6)A2F, *P*<0.001). Macro vascular-complications were also found related to its decrease. These findings, taken together, leads to the working hypothesis T2DM is related to NG1(6)A2F and might be monitored by profiling N-glycosilation of serum protein. We also found that there is an increase in the digalactosylated, diantennary glycans (NA2) in T2DM compared to CTR, which becomes more pronounced in the presence of complications (being statistically significant in males and having similar trend in females). These structural changes may occur as the result of alterations in the levels of glycosyltransferases, glycosidases, and the sugar nucleotide donors [41].

When the activity of α-1,6-fucosyltransferase 8 (FUT8) is reduced, the α(1,6)-arm monoagalacto core-α-1,6-fucosylated diantennary glycans (NG1(6)A2F) can be used by β-1,4-galactosyltransferase (βGalT) as a substrate to terminally add another galactose residue and form the digalactosylated, diantennary glycans (NA2) (see S2 Fig.). This will reduce the amount of NG1(6)A2F and increase the amount of NA2 in serum. There are some reports stating that βGalT activity is increased in diabetic patients with complications [42] thereby supporting this hypothesis. A possible reason for increased βGalT activity in diabetic patients could be an increase in UDP-glucose concentration as reported in some diabetic tissues [43]. UDP-glucose can be converted in UDP-galactose (donor substrate for βGalT) by UDP-galactose epimerase increasing the substrate available for βGalT and it's activity (see S2 Fig.).

Measuring UDP-galactose levels and glycosyltransferase activities in humans is very hard, and therefore further studies on diabetic animal model are needed to shed light on this mechanism.

We also found an important relationship among the N-glycan profile and MS. NG1(6)A2F and NG1(3)A2F levels were strongly negatively correlated with most of metabolic syndrome parameters, including waist/hip ratio, triglycerides, glycemia, glycated haemoglobin and the absolute number of neutrophils. In addition, NG1(6)A2F levels are negatively correlated with HOMA and NG1(3)A2F levels are positively correlated with HDL. These results clearly demonstrate that both N-glycans are sensitive biomarkers to this syndrome. Furthermore NG1(6)A2F and NG1(3)A2F decrease more with the increased severity of patient phenotype from the extreme “healthy” (CTR without MS) to the extreme “unhealthy” (T2DM+ with MS), suggesting a synergistic effect of the two pathological conditions on levels of these two N-glycan isomers.

Thus serum N-glycome profile can point out T2DM metabolism alterations as well as disturbances linked to MS. Changes in the glycosylation profiles of serum proteins could also be caused by changes in the clearance rate of the glycoproteins [44]. The asialoglycoprotein receptor of the liver clears glycoproteins from blood. Under stress conditions this clearance can increase thereby modifying the glycan composition in serum [45]. Metabolic syndrome could induce similar stress, enhancing this clearance capacity and modifying NG1(6)A2F and NG1(3)A2F levels.

The open question is whether N-glycosilation profile changes are consequence of T2DM and/or MS or the N-glycosilation alteration can cause or enhance the severity of T2DM, complications and MS. During e.g. diabetic microangiopathy, a generalized thickening of basement membranes occurs [46]. Glycoproteins and glycolipids form integral parts of these basement membranes so alterations in the glycosylation machinery could induce or worsen this complication. Combining glycan analysis (using DSA-FACE and high-performance liquid chromatography) with proteomics could lead to the identification of glycosylation changes, which characterise diabetes, on particular glycoproteins, providing new insights on this illness and its complications.

In conclusion, we demonstrate for the first time that N-glycan profiles of T2DM patients and/or subjects with MS show changes, that are associated also to the presence of macrovascular complications. Our data suggest that the measurement of N-glycan levels could provide a noninvasive surrogate marker for T2DM and MS. Further studies in carefully selected human cohorts and in animal model systems are needed to clarify the molecular mechanism underlying the statistical associations observed in our sample. Moreover, further studies are needed to verify whether the measurements of N-glycans can be a useful tool to assess the efficacy of therapies against T2DM and/or MS.

## Supporting Information

S1 Fig A typical desialylated N-glycan profile of human serum proteins. Each number represents a peak and indicates its molecular structure
(TIF)

S2 Fig Conversion of UDP-glucose in UDP-galactose, available for βGalT. P3, peak 3, (α(1,6)-arm monogalactosylated core-α-1,6-fucosylated diantennary glycans, NG1(6)A2F) can be used as acceptor substrate for β-1,4-galactosyltransferase (βGalT) to terminally add another galactose residue to P3 and form P5, peak 5, (digalactosylated, diantennary glycan, NA2) when there is a reduction in α-1,6-fucosyltransferase 8 (FUT8) activity (reduced core-fucose).
(TIF)

S1 Table Serum N-glycans differences between males and females. For each peak the mean and the SD are indicated. The reference group in each comparison is males. The linear regression is corrected for the age of the subjects. For each comparison, the FDR-corrected p-value (q-value) is reported if <0.05.
(DOC)
