# Supplementary figures and images for: N-Glycomic Changes in Serum Proteins in Type 2 Diabetes Mellitus Correlate with Complications and with Metabolic Syndrome Parameters
Source: PLoS One. 2015 Mar 20;10(3):e0119983. doi: 10.1371/journal.pone.0119983 (PMC4368037; doi:10.1371/journal.pone.0119983)

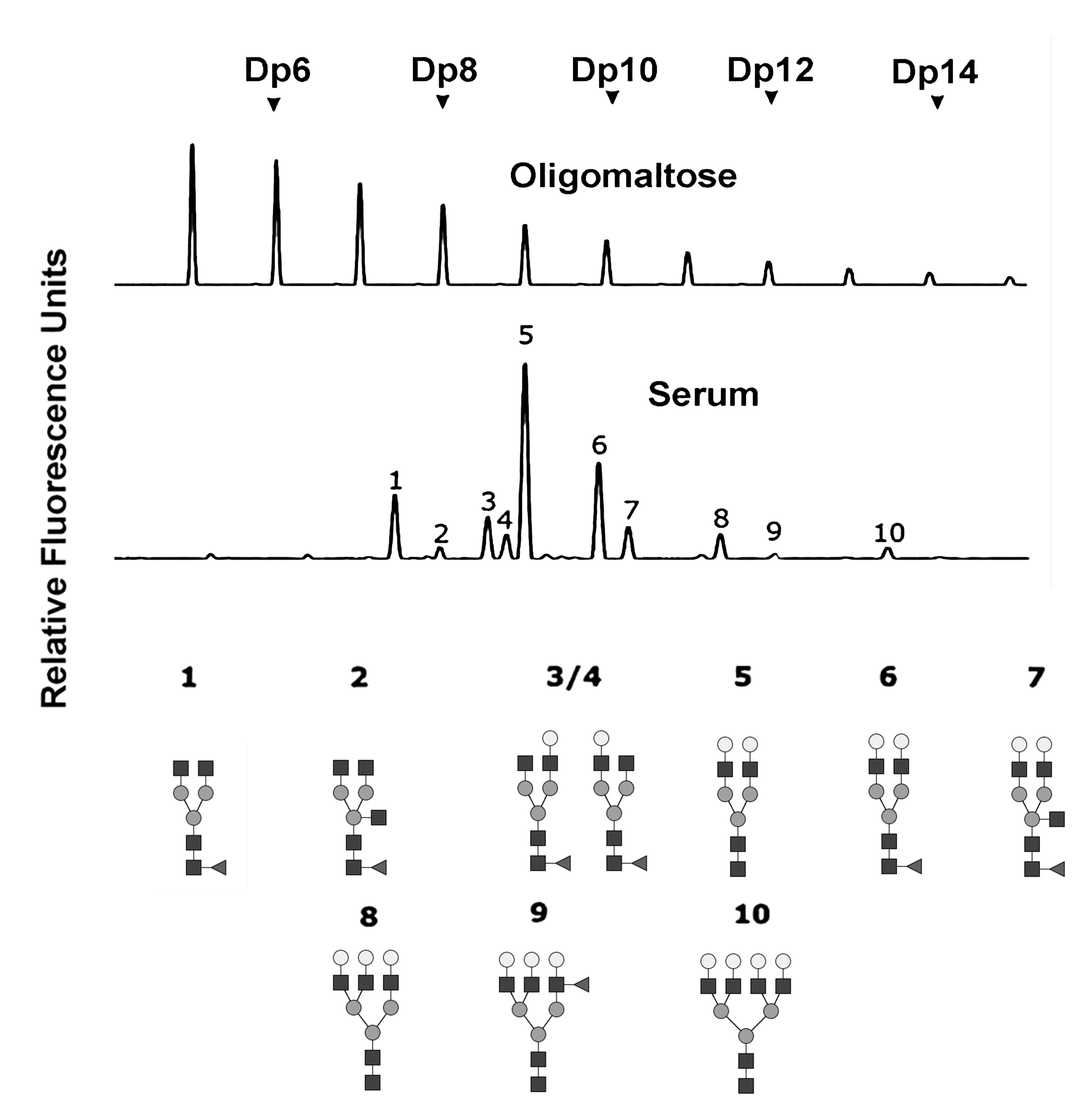

Supplement: S1 Fig — Each number represents a peak and indicates its molecular structure (TIF) [file pone.0119983.s001.tif]

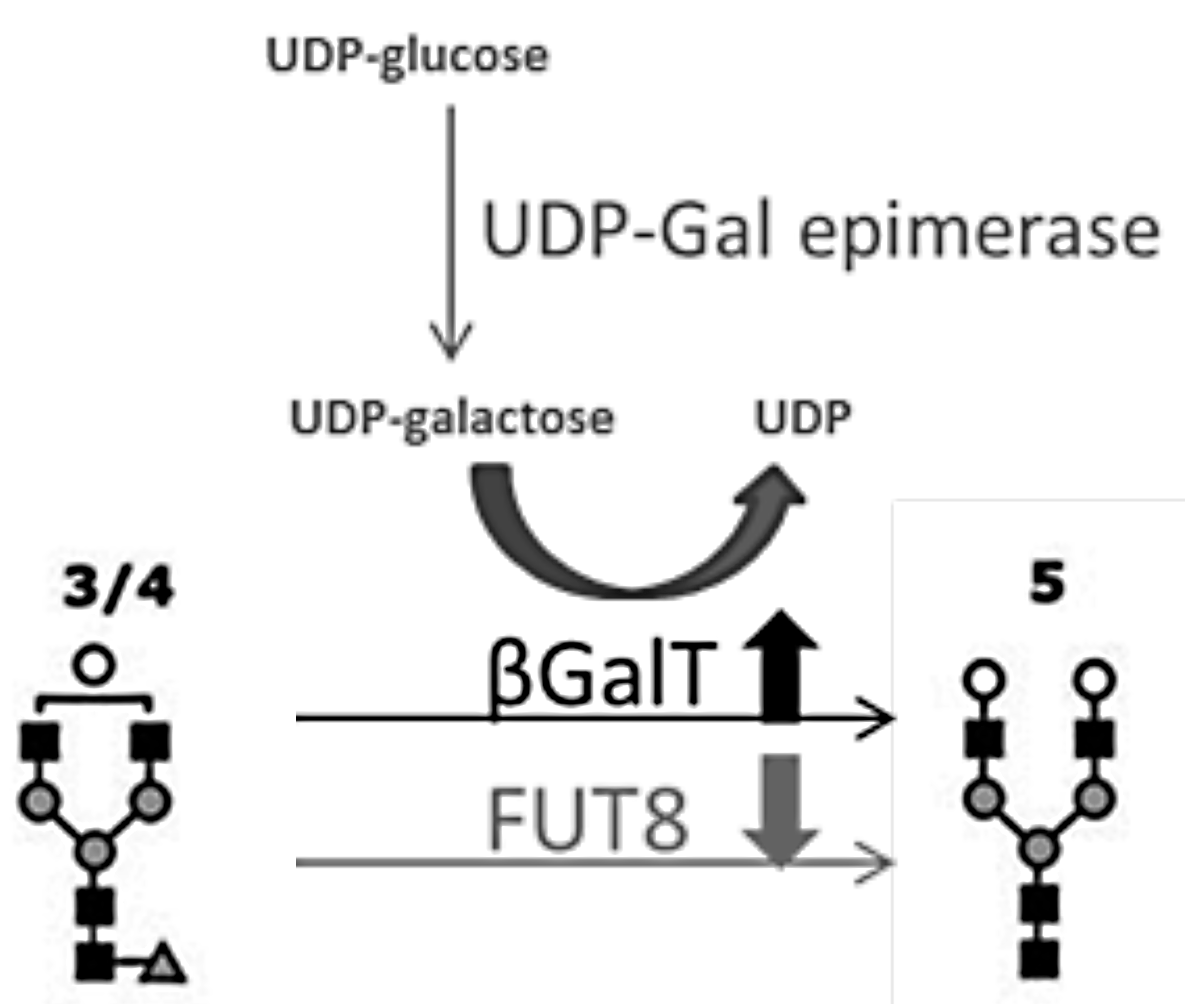

Supplement: S2 Fig — P3, peak 3, (α(1,6)-arm monogalactosylated core-α-1,6-fucosylated diantennary glycans, NG1(6)A2F) can be used as acceptor substrate for β-1,4-galactosyltransferase (βGalT) to terminally add another galactose residue to P3 and form P5, peak 5, (digalactosylated, diantennary glycan, NA2) when there is a reduction in α-1,6-fucosyltransferase 8 (FUT8) activity (reduced core-fucose). (TIF) [file pone.0119983.s002.tif]
