# Supplementary material for: N-Glycomic Changes in Serum Proteins in Type 2 Diabetes Mellitus Correlate with Complications and with Metabolic Syndrome Parameters
Source: PLoS One. 2015 Mar 20;10(3):e0119983. doi: 10.1371/journal.pone.0119983 (PMC4368037; doi:10.1371/journal.pone.0119983)
Supplement: S1 Table — For each peak the mean and the SD are indicated. The reference group in each comparison is males. The linear regression is corrected for the age of the subjects. For each comparison, the FDR-corrected p-value (q-value) is reported if <0.05. (DOC) [file pone.0119983.s003.doc]

**Table S1**.

**Table S1. Serum N-glycans differences between males and females.** For each peak the mean and the SD are indicated. The reference group in each comparison is males. The linear regression is corrected for the age of the subjects. For each comparison, the FDR-corrected p-value (q-value) is reported if <0.05.

|  | **Males** | **Females** |
| --- | --- | --- |
|  | ***Mean (SD)*** | ***Mean (SD)*** |
|  |  | ***q-value*** |
| **Peaks (structure)** |  |  |
| **P1** (NGA2F) | 9.55 (2.97) | 9.45 (3.14) |
|  |  |  |
| **P2** (NGA2FB) | 1.73 (0.62) | 1.76 (0.67) |
|  |  |  |
| **P3** (NG1[6]A2F) | 5.6 (1.42) | 5.49 (1.39) |
|  |  |  |
| **P4** (NG1[3]A2F) | 4.93 (0.94) | 4.75 (0.87) |
|  |  | ***P<0.001*** |
| **P5** (NA2) | 43.71 (4.54) | 43.67 (4.35) |
|  |  |  |
| **P6** (NA2F) | 17.7 (2.52) | 17.25 (2.79) |
|  |  | ***P<0.01*** |
| **P7** (NA2FB) | 6.06 (1.73) | 6.11 (1.47) |
|  |  |  |
| **P8** (NA3) | 6.5 (1.78) | 7.67 (1.84) |
|  |  | ***P<0.001*** |
| **P9** (NA3F) | 2.84 (1.13) | 2.17 (1.04) |
|  |  | ***P<0.001*** |
| **P10** (NA4) | 1.38 (0.46) | 1.68 (0.49) |
|  |  | ***P<0.001*** |
